# Association Between Unmet Educational Aspirations and Depressive Symptoms in Working Young Adults

**DOI:** 10.1155/da/6025004

**Published:** 2026-07-02

**Authors:** Woorim Kim, Soon Young Lee, Yeong Jun Ju

**Affiliations:** ^1^ Cancer Knowledge and Information Center, National Cancer Control Institute, National Cancer Center, Goyang-si, Gyeonggi-do, Republic of Korea, ncc.re.kr; ^2^ Department of Preventive Medicine and Public Health, Ajou University School of Medicine, Suwon-si, Gyeonggi-do, Republic of Korea, ajou.ac.kr; ^3^ Graduate School of Public Health, Ajou University, Suwon-si, Gyeonggi-do, Republic of Korea, ajou.ac.kr; ^4^ Gyeonggi Center for Hypertension and Diabetes, Ajou University, Suwon-si, Gyeonggi-do, Republic of Korea, ajou.ac.kr

**Keywords:** depression, education, educational aspirations, unmet need

## Abstract

**Background:**

Depression poses a significant burden among young adults, making it crucial to investigate factors associated with depressive symptoms. This study examined the association between unmet educational aspirations and depressive symptoms in working young adults.

**Methods:**

Data from the 2021 Seoul Young Adult Panel Study (SYPS) were used. Depressive symptoms were measured using the Center for Epidemiologic Studies Depression (CES‐D 11) scale. The association between unmet educational aspirations and depressive symptoms was analyzed using multivariable linear regression analysis. A subgroup analysis was conducted based on job satisfaction level.

**Results:**

Of 3402 participants, 392 (11.5%) reported unmet educational aspirations for personal reasons and 412 (12.1%) for circumstantial reasons. Compared to individuals without unmet educational aspirations, those with unmet educational aspirations due to personal (*β*: 0.77, *p* = 0.017) and circumstantial (*β*: 2.17, *p* < 0.001) reasons had higher CES‐D 11 scores, indicating higher levels of depressive symptoms. This increase was particularly pronounced among participants who reported circumstantial unmet educational aspirations and had low job satisfaction.

**Conclusions:**

Young adults with unmet educational aspirations, whether for personal or circumstantial reasons, showed higher levels of depressive symptoms. The findings suggest the importance of considering educational aspirations in addressing the mental health of young adults.

## 1. Introduction

Depression is recognized as a major global public health concern and ranks as a leading cause of years lived with disability [1]. In South Korea, depression imposes a significant burden, particularly in terms of disability‐adjusted life years (DALYs), with its prevalence steadily increasing and reaching ~6.1% of the adult population [2–4]. Considering the strong association between depression and suicide risk, this issue is particularly urgent in South Korea, which has the highest suicide rate among OECD countries [5, 6]. Approximately 60% of suicide cases are linked to mood disorders, including major depressive disorders [7]. Furthermore, younger adults have been identified as being at greater risk of developing depression, highlighting the importance of identifying contributing factors in this demographic group [2].

A range of socioeconomic factors, such as education, have been identified as determinants of depression risk [8]. Research has shown that lower levels of education can increase various social risks, including lower socioeconomic status and a lack of effective coping strategies for managing stress, both of which can exacerbate mental health problems [9, 10]. Additionally, educational attainment is closely linked to health literacy, which is a key factor in accessing healthcare services and using medications properly [11]. Given the well‐established connection between education and mental health and considering that education equips individuals with both the skills necessary for employment and the cognitive tools to address mental health challenges, there is reason to believe that unmet educational aspirations may be associated with depressive symptoms in working adults [12, 13]. However, studies specifically investigating the health effects of unmet educational aspirations remain scarce.

The purpose of this study was to examine the relationship between unmet educational aspirations and depressive symptoms among economically active young adults aged 19–36. Unmet educational aspirations were further classified into personal and circumstantial categories. Our hypothesis posited that individuals with unmet educational aspirations due to circumstantial reasons would exhibit higher levels of depressive symptoms. Additionally, a subgroup analysis based on job satisfaction was conducted.

## 2. Methods

### 2.1. Data and Study Population

This study utilized data from the 2021 Seoul Young Adult Panel Study (SYPS). The sample consists of adults aged 19–36 registered as residing in Seoul, selected proportionally based on sex, age, and region (district) using probability proportional to size sampling [14]. This method ensures that the selected sample is representative of Seoul’s population. Selected individuals were asked whether they agreed to participate, and the survey was conducted annually by the Seoul Institute using the computer‐assisted web interviewing (CAWI) and computer‐assisted mobile interviewing (CAMI) methods [14]. This study included individuals aged between 19 and 36 years who reported having a job and earning an income. Among the initial study population of 3409 employed young adults, we excluded two individuals with missing information on the income level and five individuals with missing data on wealth. A total of 3402 participants were therefore included in the final study population.

### 2.2. Outcome Measure

Depressive symptoms were the primary outcome of interest, assessed using the Korean version of the Center for Epidemiologic Studies Depression (CES‐D 11) scale. The CES‐D 11 measures depressive symptoms experienced over the past week on a four‐point Likert scale across 11 items. The scale ranges from 0 to 33 and can be used to detect depressive symptoms. The reliability and validity of the Korean version have been confirmed in previous research [15].

### 2.3. Independent Variables

The key independent variable in this study was unmet educational aspirations. This was measured through self‐reported responses to the question: “Do you believe you received the level of education you desired?” Responses were categorized as either “yes” or “no.” Participants who answered “no” were further asked to specify the reason for their unmet educational goals from a predefined list of response options.

For analytic purposes, the reported reasons were grouped into two categories based on the nature of the reported barriers (Table S1). Reasons reflecting the respondent’s life circumstances, including financial difficulties, discrimination from family members, caregiving responsibilities, and physical disability or disease, were classified as circumstantial reasons. The remaining reasons, including failure to pass the entrance examination and geographic distance from educational institutions, were classified as personal reasons. This classification is an operational grouping and does not imply voluntariness, individual blame, or moral accountability for unmet educational aspirations.

Covariates included in the analysis were age, sex, education level, income level, wealth status, job type, job classification, marital status, financial independence, economic hardship, job satisfaction, education‐job alignment, and subjective health status. Wealth was defined as including real estate, financial assets, and virtual currency. Economic hardship was assessed by asking participants whether they had experienced difficulties, such as the inability to visit a hospital due to financial constraints, trouble paying housing or utility bills, or having a household member with credit card delinquency.

### 2.4. Statistical Analysis

*T*‐tests and analysis of variance (ANOVA) were conducted to examine the general characteristics of the sample and compare mean CES‐D 11 scores (mean ± standard deviation [SD]). The association between unmet educational aspirations and depressive symptoms was analyzed using multivariable linear regression adjusting for all covariates. To evaluate the robustness of the findings, we conducted sensitivity analyses using several alternative modeling approaches: (1) robust standard errors to address potential heteroscedasticity, (2) robust regression to reduce the influence of outliers, (3) a log‐transformed model to account for the right‐skewed distribution of the outcome variable, and (4) quantile regression to examine associations across the conditional outcome distribution. A subgroup analysis was also performed, stratified by job satisfaction. All analyses were adjusted for covariates, and statistical significance was set at *p* < 0.05 for two‐sided tests. All analyses were conducted using SAS software (Version 9.4, SAS Institute, Cary, NC, USA). Additionally, to assess whether conceptual overlap between the independent variable and certain covariates affected the results, we conducted a sensitivity analysis by re‐estimating the model after excluding wealth status, economic hardship, and subjective health status. Variance inflation factors (VIFs), tolerance, and condition indices were also computed to evaluate multicollinearity (Tables S3–S5).

### 2.5. Ethical Considerations

This study adhered to the ethical standards outlined by relevant national and institutional committees on human experimentation as well as the Declaration of Helsinki of 1975. The data used were anonymized and did not contain any personal identifiers, as all such information was fully deidentified prior to release. According to Article 2.2 of the Enforcement Rule of the Bioethics and Safety Act in Korea, the use of these data is exempt from institutional review board (IRB) review. Given the use of anonymized public data, this exemption was confirmed by the IRB of the Seoul Institute, and the requirement for informed consent was waived.

## 3. Results

The general characteristics of the study population are presented in Table 1. Among the 3402 participants, 392 individuals (11.5%) reported having unmet educational aspirations due to personal reasons, and 412 individuals (12.1%) reported circumstantial reasons. The mean CES‐D 11 scores were higher among participants with unmet educational aspirations, whether due to personal reasons (mean ± SD: 7.69 ± 6.54) or circumstantial reasons (10.82 ± 8.21), compared to those without unmet educational aspirations (6.57 ± 6.31). When applying a cutoff score of 16 to indicate depressive symptoms, 13.2% of the study participants had depressive symptoms.

**Table 1:** General characteristics of the study participants.

Variables	Total		Depressive symptoms			*p*‐Value
	*N*	%	Mean	±	SD^a^	
Unmet educational aspiration						<0.001
No	2598	76.4	6.57	±	6.31	
Yes (personal reasons)	392	11.5	7.69	±	6.54	
Yes (circumstantial reasons)	412	12.1	10.82	±	8.21	
Age						0.704
19–24	802	23.6	7.16	±	6.65	
25–29	1289	37.9	7.37	±	6.78	
30–34	1267	37.2	7.10	±	6.79	
35–36	44	1.3	6.64	±	5.27	
Sex						<0.001
Male	1545	45.4	6.52	±	6.58	
Female	1857	54.6	7.78	±	6.81	
Education level						<0.001
High school or below	1024	30.1	7.51	±	7.04	
Community college	476	14.0	8.12	±	6.96	
University (undergraduate)	1685	49.5	6.81	±	6.49	
Graduate school	217	6.4	6.88	±	6.40	
Income level						<0.001
Low	854	25.1	7.23	±	6.65	
Middle low	849	25.0	8.05	±	7.10	
Middle high	949	27.9	6.92	±	6.52	
High	750	22.1	6.59	±	6.58	
Wealth status						0.133
Low	856	25.2	8.40	±	7.21	
Middle low	895	26.3	7.32	±	6.79	
Middle high	859	25.3	6.58	±	6.34	
High	792	23.3	6.49	±	6.37	
Job type						0.003
White collar	2156	63.4	6.95	±	6.43	
Pink collar	848	24.9	7.44	±	6.98	
Blue collar	398	11.7	8.15	±	7.68	
Job classification						0.003
Permanent employee	1980	58.2	6.86	±	6.43	
Precarious employee	434	12.8	7.90	±	7.03	
Part‐time or day laborer	723	21.3	7.77	±	7.17	
Self‐employed	265	7.8	7.20	±	7.12	
Marital status						0.047
Married	444	13.1	6.55	±	6.56	
Divorced, widowed, or separated	21	0.6	8.90	±	7.77	
Single	2937	86.3	7.30	±	6.75	
Financial independence						0.066
Yes	2307	67.8	7.35	±	6.85	
No	1095	32.2	6.91	±	6.49	
Economic hardship						<0.001
Low	2820	82.9	6.33	±	6.11	
Mediocre	323	9.5	10.25	±	7.50	
High	259	7.6	13.05	±	8.10	
Job satisfaction						<0.001
Mediocre	1231	36.2	7.47	±	6.58	
High	1665	48.9	5.89	±	5.94	
Low	506	14.9	10.94	±	7.99	
Education and job match						<0.001
Educational level > job skills	991	29.1	7.94	±	7.22	
Similar	2141	62.9	6.80	±	6.41	
Educational level < job skills	270	7.9	7.79	±	7.13	
Subjective health status						<0.001
Fair	1875	55.1	4.89	±	5.20	
Poor	1527	44.9	10.06	±	7.30	

Total	3402	100.0	7.21	±	6.73	

^a^SD: standard deviation.

Table 2 shows the results of the regression analysis examining the association between unmet educational aspirations and depressive symptoms. Compared to individuals without unmet educational aspirations, those with personal (*β*: 0.77, *p* = 0.017) or circumstantial (*β*: 2.17, *p* < 0.001) unmet educational aspirations exhibited significantly higher CES‐D 11 scores, indicating greater depressive symptoms. Participants with low job satisfaction also had significantly higher depression scores (*β*: 2.64, *p* < 0.001), while those with high job satisfaction exhibited lower depression scores (*β*: −0.65, *p* = 0.004). These results remained consistent across all alternative models, including robust standard errors, robust regression, the log‐transformed model, and quantile regression, indicating that the main findings were robust to different modeling approaches (Table S2). Furthermore, the VIFs for all variables were below 10 (maximum VIF, 3.02), all tolerance values exceeded 0.10 (minimum tolerance, 0.33), and the maximum condition index was 18.34, well below the threshold of 30, indicating that multicollinearity was not a concern (Tables S3 and S4). When the model was re‐estimated after excluding wealth status, economic hardship, and subjective health status, the associations remained consistent, with the estimate for circumstantial reasons showing a larger magnitude (*β*: 3.52, *p* < 0.001), suggesting that the fully adjusted model provides a conservative estimate of the association (Table S5).

**Table 2:** The association between unmet educational aspirations and depressive symptoms.

Variables	Depressive symptoms			
	Adjusted‐*β*	SE^a^	*p*‐Value	95% CI^b^
Unmet educational aspiration				
No	Ref.			
Yes (personal reasons)	0.77	0.32	0.017	(0.14, 1.40)
Yes (circumstantial reasons)	2.17	0.38	<0.001	(1.42, 2.91)
Age				
19–24	Ref.			
25–29	0.27	0.33	0.416	(−0.38, 0.93)
30–34	0.21	0.38	0.589	(−0.54, 0.96)
35–36	0.26	0.72	0.720	(−1.16, 1.68)
Sex				
Male	Ref.			
Female	0.95	0.22	<0.001	(0.52, 1.37)
Education level				
High school or below	Ref.			
Community college	−0.02	0.38	0.957	(−0.76, 0.72)
University (undergraduate)	−0.38	0.31	0.230	(−0.99, 0.24)
Graduate school	−0.23	0.52	0.649	(−1.24, 0.78)
Income level				
Low	Ref.			
Middle low	0.57	0.33	0.082	(−0.07, 1.21)
Middle high	0.39	0.33	0.238	(−0.26, 1.04)
High	0.84	0.38	0.027	(0.10, 1.58)
Wealth status				
Low	Ref.			
Middle low	−0.55	0.30	0.072	(−1.14, 0.05)
Middle high	−0.74	0.33	0.023	(−1.38, −0.11)
High	−0.84	0.37	0.021	(−1.56, −0.13)
Job type				
White collar	Ref.			
Pink collar	0.10	0.28	0.724	(−0.45, 0.65)
Blue collar	0.58	0.39	0.136	(−0.18, 1.34)
Job classification				
Permanent employee	Ref.			
Precarious employee	0.40	0.35	0.252	(−0.28, 1.07)
Part‐time or day laborer	0.44	0.33	0.184	(−0.21, 1.09)
Self‐employed	0.01	0.43	0.986	(−0.84, 0.85)
Marital status				
Married	Ref.			
Divorced, widowed, or separated	0.63	1.51	0.678	(−2.34, 3.60)
Single	0.50	0.33	0.135	(−0.16, 1.16)
Financial independence				
No	Ref.			
Yes	0.22	0.24	0.371	(−0.26, 0.70)
Economic hardship				
Low	Ref.			
Mediocre	2.27	0.42	<0.001	(1.45, 3.09)
High	4.11	0.51	<0.001	(3.10, 5.11)
Job satisfaction				
Mediocre	Ref.			
Low	2.64	0.37	<0.001	(1.92, 3.37)
High	−0.65	0.23	0.004	(−1.10, −0.21)
Education and job match				
Similar	Ref.			
Educational level > job skills	−0.16	0.26	0.528	(−0.67, 0.34)
Educational level < job skills	0.41	0.42	0.321	(−0.40, 1.23)
Subjective health status				
Fair	Ref.			
Poor	3.96	0.22	<0.001	(3.53, 4.40)

^a^SE: standard error.

^b^95% CI: 95% confidence interval.

The results of the subgroup analysis based on job satisfaction are presented in Table 3. The association between unmet educational aspirations and depressive symptoms was maintained regardless of the level of job satisfaction. However, the increase in depression scores was particularly pronounced among participants who reported circumstantial unmet educational aspirations and had low job satisfaction (*β*: 3.75, *p* < 0.001).

**Table 3:** Results of the subgroup analysis conducted based on job satisfaction level.

Variables		Depressive Symptoms			
		Adjusted‐*β*	SE^a^	*p*‐Value	95% CI^b^
Job satisfaction					
Low	Unmet educational aspiration				
	No	1.00			
	Yes (personal reasons)	0.09	0.99	0.931	(−1.87, 2.04)
	Yes (circumstantial reasons)	3.75	0.91	<0.001	(1.96, 5.54)
Mediocre	Unmet educational aspiration				
	No	1.00			
	Yes (personal reasons)	0.74	0.54	0.172	(−0.32, 1.80)
	Yes (circumstantial reasons)	1.40	0.61	0.021	(0.22, 2.59)
High	Unmet educational aspiration				
	No	1.00			
	Yes (personal reasons)	0.92	0.43	0.034	(0.07, 1.77)
	Yes (circumstantial reasons)	1.97	0.58	0.001	(0.84, 3.10)

^a^SE: standard error.

^b^95% CI: 95% confidence interval.

## 4. Discussion

This study examined the association between unmet educational aspirations and depressive symptoms in working young adults. The findings demonstrated that individuals with unmet educational aspirations, whether due to personal or circumstantial reasons, had higher depression scores, indicating more severe depressive symptoms. This association was especially pronounced among those who experienced circumstantial unmet educational aspirations. Furthermore, while this trend was evident regardless of the level of job satisfaction, the increase in depression scores was most significant among individuals with circumstantial unmet educational aspirations and low job satisfaction.

Previous research on the relationship between educational attainment and depressive symptoms consistently reports an association between higher levels of education and better mental health outcomes [16]. A Swedish study found that lower educational attainment was correlated with a higher incidence of depression in young adulthood, emphasizing the importance of enhancing educational attainment to address the mental health needs of this demographic [17]. Similarly, a study of Chinese women demonstrated that lower educational attainment increased the likelihood of developing major depressive disorder [18]. A Korean study also identified low educational attainment as a potential risk factor for depression, while a longitudinal study of the Indonesian population revealed the long‐term impact of educational attainment on depression in later life [10, 19]. This study contributes to the existing literature by demonstrating that unmet educational aspirations are associated with higher levels of depressive symptoms.

These findings may be explained by the role of educational expectations, which have been linked to the risk of depression, similarly to educational aspirations. Social constraints that limit educational opportunities have been reported to affect an individual’s health [20]. Educational expectations refer to the level of education an individual anticipates achieving and reflect structural factors such as socioeconomic status and parental support, both of which influence academic outcomes [21]. Since these external factors are beyond an individual’s control, those with higher educational expectations are often better positioned with the socioeconomic resources necessary for maintaining health, whereas individuals with unmet expectations may experience heightened depressive symptoms [22, 23]. Given that the reasons for unmet educational aspirations identified in this study, such as financial hardship and family‐based gender discrimination, align with factors underlying educational expectations, it is plausible that unmet educational aspirations contributed to the increased levels of depressive symptoms observed in this study.

The association between unmet educational aspirations and depressive symptoms persisted regardless of job satisfaction, with the magnitude being particularly pronounced among individuals with low job satisfaction. Job satisfaction refers to the emotional state resulting from an individual’s evaluation of their occupation, typically influenced by factors such as income, work environment, and working hours [24, 25]. It is recognized as a key indicator of workplace mental health and has been associated with depression in economically active adults [26, 27]. For example, a meta‐analysis demonstrated a correlation between job satisfaction and depressive symptoms among workers [28]. Furthermore, studies have identified job satisfaction as a predictor of developing depression [29]. Given that educational aspirations and occupation are critical indicators of socioeconomic status, with education reflecting access to resources and occupation representing social standing, and both have been independently linked to depressive symptoms, individuals with circumstantial unmet educational aspirations and low job satisfaction may be particularly susceptible to depressive symptoms [30].

### 4.1. Limitations

This study has several limitations. First, it employed a cross‐sectional design, as unmet educational aspirations were measured only in the 2021 SYPS data, limiting the ability to establish causality. The cross‐sectional nature of the data implies that reverse causation is plausible and that depressive symptoms may influence the study participants’ perception of having unmet educational aspirations. Future studies using longitudinal data are needed to address this limitation and provide further insights. Second, the generalizability of the findings may be constrained, as the SYPS sample includes only individuals residing in Seoul, the capital city of Korea. However, the results remain meaningful, given that over one‐fifth of the studied age group is reported to reside in Seoul [31, 32]. Third, while various work‐related factors were included as covariates in the analysis, certain characteristics, such as shift work, could not be accounted for due to data limitations. Furthermore, potentially important confounders, such as a history of adversities or trauma during childhood, adolescence, or early adulthood, were not included due to data limitations. Fourth, the classification of reasons for unmet educational aspirations into personal and circumstantial categories was based on an operational grouping of predefined survey response options. The boundary between the two categories is not absolute as some reasons may be interpreted differently depending on the social and cultural context. For instance, failure to pass an entrance examination may reflect not only individual academic preparedness but also structural inequalities in access to educational resources, and geographic distance from educational institutions may be intertwined with financial constraints or family obligations. Future research using more granular measures of educational barriers across diverse cultural settings would help refine this classification. Despite these limitations, this study is significant as it is the first to examine the association between unmet educational aspirations, job satisfaction, and depressive symptoms in economically active young adults using large‐scale data.

## 5. Conclusion

Economically active young adults with unmet educational aspirations, whether due to personal or circumstantial reasons, exhibited higher levels of depressive symptoms. This association was particularly pronounced among individuals with circumstantial unmet educational aspirations who also reported low job satisfaction. These findings highlight the potential significance of addressing educational aspirations when considering the mental health of young adults.

## Author Contributions

**Woorim Kim**: conceptualization, methodology, formal analysis, validation, writing – original draft, writing – review and editing. **Soon Young Lee**: conceptualization, validation, writing – original draft. **Yeong Jun Ju**: conceptualization, methodology, formal analysis, data curation, validation, writing – original draft, writing – review and editing, visualization.

## Funding

This work was supported by the National Research Foundation of Korea (NRF) grant funded by the Korea government (Ministry of Science and ICT) (Grant RS‐2022‐NR072242).

## Disclosure

All authors read and approved the final version of the manuscript. The funding source had no involvement in the study design, data analysis and interpretation, writing of the manuscript, or in the decision to submit the manuscript for publication.

## Conflicts of Interest

The authors declare no conflicts of interest.

## Supporting Information

Additional supporting information can be found online in the Supporting Information section.

## Supporting information


**Supporting Information** Table S1: Presents the categorization of reasons for having unmet educational aspirations. Table S2: Presents the association between unmet educational aspirations and depressive symptoms across alternative modeling approaches. Table S3: Presents the variance inflation factors and tolerance values for covariates included in the multivariable linear regression model. Table S4: Presents the collinearity diagnostics, including eigenvalues and condition indices, for the multivariable linear regression model. Table S5: Presents a sensitivity analysis of the association between unmet educational aspirations and depressive symptoms after excluding wealth status, economic hardship, and subjective health status.

## Data Availability

Data will be made available upon request. The dataset is available on the Seoul Young Adult Panel Study website (http://syps.si.re.kr/homepage).
