# Supplementary material for: Association Between Unmet Educational Aspirations and Depressive Symptoms in Working Young Adults
Source: Depress Anxiety. 2026 Jul 2;2026:6025004. doi: 10.1155/da/6025004 (PMC13328832; doi:10.1155/da/6025004)
Supplement: Supplementary file 1 — Supporting Information Table S1: Presents the categorization of reasons for having unmet educational aspirations. Table S2: Presents the association between unmet educational aspirations and depressive symptoms across alternative modeling approaches. Table S3: Presents the variance inflation factors and tolerance values for covariates included in the multivariable linear regression model. Table S4: Presents the collinearity diagnostics, including eigenvalues and condition indices, for the multivariable linear regression model. Table S5: Presents a sensitivity analysis of the association between unmet educational aspirations and depressive symptoms after excluding wealth status, economic hardship, and subjective health status. [file DA-2026-6025004-s001.docx]

**Supplementary Table S1. Categorization of reasons for having educational unmet aspirations**

| **Reason** | **Categorization** |
| --- | --- |
| Failure to pass the entrance exam | Personal reasons |
| Long travel distance from home |  |
| Financial difficulties | Circumstantial reasons |
| Discrimination from family (sexual discrimination, low recognition) |  |
| Physical disability or disease |  |
| The need to take care of family members or housework |  |

| **Supplementary Table S2. The association between unmet educational aspirations and depressive symptoms across alternative modeling approaches** | | | |
| --- | --- | --- | --- |
| **Variables** | **Depressive Symptoms** | | |
|  | **Adjusted-β** | **SE*** | **p-value** |
| **Robust standard errors** | | | |
| **Unmet educational aspiration** |  |  |  |
| No | Ref. |  |  |
| Yes (personal reasons) | 0.83 | 0.32 | 0.009 |
| Yes (circumstantial reasons) | 2.09 | 0.37 | <.001 |
| **Log-transformed model** | | | |
| **Unmet educational aspiration** |  |  |  |
| No | Ref. |  |  |
| Yes (personal reasons) | 0.16 | 0.05 | 0.001 |
| Yes (circumstantial reasons) | 0.26 | 0.05 | <.001 |
| **Robust regression model** | | | |
| **Unmet educational aspiration** |  |  |  |
| No | Ref. |  |  |
| Yes (personal reasons) | 0.87 | 0.31 | 0.005 |
| Yes (circumstantial reasons) | 1.54 | 0.37 | <.001 |
| **Quantile regression model** | | | |
| **Unmet educational aspiration** |  |  |  |
| No | Ref. |  |  |
| Yes (personal reasons) | 0.89 | 0.42 | 0.036 |
| Yes (circumstantial reasons) | 1.65 | 0.43 | <.001 |

** SE: Standard Error*

| **Supplementary Table S3. Variance inflation factors and tolerance values for covariates included in the multivariable linear regression model** | | | |
| --- | --- | --- | --- |
| **Variables** | **Variance Inflation** | **Tolerance** |  |
| **Unmet educational aspiration** |  |  |  |
| No | Ref. |  |  |
| Yes (personal reasons) | 1.05 | 0.96 |  |
| Yes (circumstantial reasons) | 1.10 | 0.91 |  |
| **Age** |  |  |  |
| 19~24 | Ref. |  |  |
| 25~29 | 2.39 | 0.42 |  |
| 30~34 | 3.02 | 0.33 |  |
| 35~36 | 1.06 | 0.94 |  |
| **Sex** |  |  |  |
| Male | Ref. |  |  |
| Female | 1.10 | 0.91 |  |
| **Education level** |  |  |  |
| High school or below | Ref. |  |  |
| Community college | 1.49 | 0.67 |  |
| University (undergraduate) | 2.18 | 0.46 |  |
| Graduate school | 1.42 | 0.71 |  |
| **Income level** |  |  |  |
| Low | Ref. |  |  |
| Middle low | 1.89 | 0.53 |  |
| Middle high | 2.29 | 0.44 |  |
| High | 2.39 | 0.42 |  |
| **Wealth status** |  |  |  |
| Low | Ref. |  |  |
| Middle low | 1.56 | 0.64 |  |
| Middle high | 1.78 | 0.56 |  |
| High | 2.08 | 0.48 |  |
| **Job type** |  |  |  |
| White collar | Ref. |  |  |
| Pink collar | 1.40 | 0.72 |  |
| Blue collar | 1.24 | 0.81 |  |
| **Job classification** |  |  |  |
| Permanent employee | Ref. |  |  |
| Precarious employee | 1.18 | 0.85 |  |
| Part-time or day laborer | 1.68 | 0.60 |  |
| Self-employed | 1.18 | 0.85 |  |
| **Marital status** |  |  |  |
| Married | Ref. |  |  |
| Divorced, widowed, or separated | 1.06 | 0.94 |  |
| Single | 1.23 | 0.81 |  |
| **Financial independence** |  |  |  |
| No | Ref. |  |  |
| Yes | 1.21 | 0.83 |  |
| **Economic hardship** |  |  |  |
| Low | Ref. |  |  |
| Mediocre | 1.07 | 0.93 |  |
| High | 1.12 | 0.89 |  |
| **Job satisfaction** |  |  |  |
| Mediocre | Ref. |  |  |
| Low | 1.26 | 0.80 |  |
| High | 1.32 | 0.76 |  |
| **Education and job match** |  |  |  |
| Similar | Ref. |  |  |
| Educational level > job skills | 1.24 | 0.80 |  |
| Educational level < job skills | 1.06 | 0.94 |  |
| **Subjective health status** |  |  |  |
| Fair | Ref. |  |  |
| Poor | 1.12 | 0.89 |  |

| **Supplementary Table S4. Collinearity diagnostics: eigenvalues and condition indices for the multivariable linear regression model** | | | |
| --- | --- | --- | --- |
| **Number** | **Eigenvalue** | **Condition Index*** |  |
| 1 | 9.06 | 1.00 |  |
| 2 | 1.94 | 2.16 |  |
| 3 | 1.31 | 2.63 |  |
| 4 | 1.26 | 2.68 |  |
| 5 | 1.12 | 2.84 |  |
| 6 | 1.08 | 2.90 |  |
| 7 | 1.05 | 2.94 |  |
| 8 | 1.04 | 2.95 |  |
| 9 | 1.03 | 2.96 |  |
| 10 | 0.97 | 3.06 |  |
| 11 | 0.95 | 3.08 |  |
| 12 | 0.94 | 3.10 |  |
| 13 | 0.93 | 3.12 |  |
| 14 | 0.92 | 3.14 |  |
| 15 | 0.86 | 3.25 |  |
| 16 | 0.84 | 3.29 |  |
| 17 | 0.77 | 3.43 |  |
| 18 | 0.71 | 3.56 |  |
| 19 | 0.66 | 3.72 |  |
| 20 | 0.62 | 3.81 |  |
| 21 | 0.49 | 4.32 |  |
| 22 | 0.48 | 4.33 |  |
| 23 | 0.46 | 4.43 |  |
| 24 | 0.34 | 5.16 |  |
| 25 | 0.31 | 5.42 |  |
| 26 | 0.24 | 6.16 |  |
| 27 | 0.19 | 6.83 |  |
| 28 | 0.15 | 7.78 |  |
| 29 | 0.14 | 7.94 |  |
| 30 | 0.10 | 9.42 |  |
| 31 | 0.03 | 18.34 |  |

**A condition index exceeding 30 suggests potentially problematic multicollinearity*

| **Supplementary Table S5. Sensitivity analysis: association between unmet educational aspirations and depressive symptoms after excluding wealth status, economic hardship, and subjective health status** | | | | |
| --- | --- | --- | --- | --- |
| **Variables** | **Depressive symptoms** | | | |
|  | **Adjusted-β** | **SE*** | **P-value** | **95% CI**** |
| **Unmet educational aspiration** |  |  |  |  |
| No | Ref. |  |  |  |
| Yes (personal reasons) | 0.77 | 0.35 | 0.025 | (0.10 – 1.45) |
| Yes (circumstantial reasons) | 3.52 | 0.41 | <.001 | (2.72 – 4.32) |
| **Age** |  |  |  |  |
| 19~24 | Ref. |  |  |  |
| 25~29 | 0.57 | 0.36 | 0.113 | (-0.13 – 1.27) |
| 30~34 | 0.64 | 0.40 | 0.109 | (-0.14 – 1.42) |
| 35~36 | -0.01 | 0.83 | 0.986 | (-1.64 – 1.61) |
| **Sex** |  |  |  |  |
| Male | Ref. |  |  |  |
| Female | 1.29 | 0.23 | <.001 | (0.84 – 1.75) |
| **Education level** |  |  |  |  |
| High school or below | Ref. |  |  |  |
| Community college | 0.14 | 0.41 | 0.736 | (-0.66 – 0.94) |
| University (undergraduate) | -0.81 | 0.34 | 0.016 | (-1.47 – -0.15) |
| Graduate school | -0.60 | 0.55 | 0.275 | (-1.68 – 0.48) |
| **Income level** |  |  |  |  |
| Low | Ref. |  |  |  |
| Middle low | 0.54 | 0.35 | 0.119 | (-0.14 – 1.23) |
| Middle high | 0.02 | 0.35 | 0.959 | (-0.67 – 0.71) |
| High | 0.29 | 0.39 | 0.462 | (-0.48 – 1.05) |
| **Job type** |  |  |  |  |
| White collar | Ref. |  |  |  |
| Pink collar | 0.23 | 0.31 | 0.459 | (-0.38 – 0.84) |
| Blue collar | 0.83 | 0.41 | 0.043 | (0.02 – 1.64) |
| **Job classification** |  |  |  |  |
| Permanent employee | Ref. |  |  |  |
| Precarious employee | 0.59 | 0.37 | 0.106 | (-0.13 – 1.31) |
| Part-time or day laborer | 0.78 | 0.36 | 0.029 | (0.08 – 1.49) |
| Self-employed | 0.33 | 0.46 | 0.478 | (-0.57 – 1.22) |
| **Marital status** |  |  |  |  |
| Married | Ref. |  |  |  |
| Divorced, widowed, or separated | 1.41 | 1.47 | 0.334 | (-1.46 – 4.29) |
| Single | 0.49 | 0.35 | 0.165 | (-0.20 – 1.19) |
| **Financial independence** |  |  |  |  |
| No | Ref. |  |  |  |
| Yes | 0.61 | 0.26 | 0.021 | (0.09 – 1.12) |
| **Job satisfaction** |  |  |  |  |
| Mediocre | Ref. |  |  |  |
| Low | 3.00 | 0.40 | <.0001 | (2.22 – 3.78) |
| High | -1.45 | 0.24 | <.0001 | (-1.93 – -0.98) |
| **Education and job match** |  |  |  |  |
| Similar | Ref. |  |  |  |
| Educational level > job skills | -0.07 | 0.28 | 0.803 | (-0.63 – 0.48) |
| Educational level < job skills | 1.01 | 0.43 | 0.019 | (0.17 – 1.85) |

** SE: Standard Error; **95% CI: 95% Confidence Interval*
